# Muscle and Subcutaneous Fatty Acid Composition and the Evaluation of Ageing Time on Meat Quality Parameters of Hispano-Bretón Horse Breed

**DOI:** 10.3390/ani11051421

**Published:** 2021-05-15

**Authors:** Lorea R. Beldarrain, Lara Morán, Miguel Ángel Sentandreu, Kizkitza Insausti, Luis Javier R. Barron, Noelia Aldai

**Affiliations:** 1 Lactiker Research Group, Department of Pharmacy & Food Sciences, University of the Basque Country (UPV/EHU), 01006 Vitoria-Gasteiz, Spain; lorea.rivera@ehu.eus (L.R.B.); lara.moran@ehu.eus (L.M.); luisjavier.rbarron@ehu.eus (L.J.R.B.); 2 Instituto de Agroquímica y Tecnología de Alimentos (CSIC), 46980 Paterna, Spain; ciesen@iata.csic.es; 3 IS-FOOD, Research Institute for Innovation & Sustainable Development in Food Chain, Public University of Navarre (UPNA), 31006 Pamplona, Spain; kizkitza.insausti@unavarra.es

**Keywords:** ageing, color, equine, foal, intramuscular fat, texture

## Abstract

**Simple Summary:**

Horse meat; even though is still not popular in most countries; its consumption is slowly increasing and has the potential to become an alternative future red meat. However; research is still insufficient and a deeper understanding of its nutritional and physicochemical characteristics would be beneficial for the horse meat industry. The capacity of horses to efficiently uptake polyunsaturated fatty acids into their tissues has been reported; but detailed knowledge about horse meat fatty acid composition is limited. The present work provides a comprehensive fatty acid composition analysis of subcutaneous and muscle tissues from semiextensively reared Hispano-Breton horses; results indicated that finishing on a high-grain diet limited muscle n-3 accumulation. In addition; the evolution of physicochemical quality parameters such as pH, instrumental color, texture and cook loss were thoroughly studied during vacuum ageing (0, 7, 14 and 21 days), and the conclusion was that an ageing period between 7 and 14 days would be recommended for an optimum horse meat quality. The reasons for this recommendation were that tenderness increased during the first two weeks and then stayed stable and that visual properties deteriorated after 14 days. Overall; these results will help to standardize *post mortem* practices to obtain a homogeneous final horse meat quality.

**Abstract:**

A full-randomized block design was used for the study of the FA composition and meat quality parameters, considering ageing time as a split-plot factor. Chemical and fatty acid composition of steaks (longissimus thoracis and lumborum muscle) from 15 month old semiextensively reared Hispano-Bretón horses were characterized (day 0), and the effect of vacuum ageing (0, 7, 14 and 21 days) on several meat quality parameters (pH, instrumental color and texture and cook loss) was determined. The average fat content of horse loin was 3.31%, and the n-3 polyunsaturated fatty acid content, although higher than in ruminant meats, suggested that the finishing on a high-grain diet limited muscle n-3 accumulation. Results revealed that ageing affected all meat quality measurements; color started to turn brownish at 14 days of ageing, with a decrease in redness but not in yellowness. Tenderness improved during the first two weeks, and the Warner-Bratzler shear force scores showed that meat aged for 7 days could be considered as ‘intermediate tender’. Under the present study conditions, an ageing period between 7 and 14 days is recommended for an optimum horse meat quality.

## 1. Introduction

Horse meat, due to religious and/or cultural reasons, is still not popular [1,2]. Nevertheless, its consumption is slowly increasing in several countries due to its recognized nutritional value, mainly related to grazing systems [3]. Even though the effects of horse meat consumption on human health have not been widely studied [4], several investigations have addressed the interest of its nutritional properties with special attention on its lipid composition, which is a direct consequence of the equine digestive physiology [5,6,7,8]. Horses are non-ruminant herbivores and hindgut fermenters that efficiently digest and absorb the major part of dietary lipids in the foregut, before reaching microbial metabolism in the hindgut [9,10]. Thereupon, lipid composition of horse muscle tissue is generally characterized by high levels of polyunsaturated fatty acids (PUFAs) [11] and low levels of branched-chain fatty acids (BCFAs), *trans*-fatty acids and conjugated linolenic acids (CLA); that are all associated with rumen metabolism and are, therefore, more abundant in ruminant derived products [12,13,14]. Recent investigations have opened an interesting research field describing plasmalogenic lipids in horse tissues [5,15] that deserve further research due to their implications in human health [16,17]. Regarding horse meat quality, a number of studies have been performed in several breeds slaughtered at different ages [18,19,20,21,22] but there is still no general agreement concerning the *post mortem* ageing of horse meat and its effect on the final meat quality [23].

In this context, meat ageing has been commonly applied in the meat industry for decades [24]. In particular, vacuum packaging of subprimals or cuts for a certain period of cooling storage, namely wet ageing, is the most extended type of ageing [25]. This process is widely known to have a pivotal role in the improvement of meat palatability, especially tenderness [26], and variation in color [27,28], while avoiding contamination and weight loss.

The role of ageing and biochemical mechanisms involved in the improvement of meat tenderness have been widely studied [29,30], and it is well known that the level of tenderization will majorly depend on duration and temperature of the ageing process, together with other factors such as muscle type, breed and animal species [31]. Regarding color, when meat is vacuum packaged, the oxygen depleted atmosphere causes meat surface turn purple color (deoxymyoglobin) and when the package is opened and exposed to air (oxygenated), purple turns back to red color (oxymyoglobin). As the ageing process progresses, oxygen consumption of respiratory enzymes within mitochondria decreases, resulting in more oxygen available to bind myoglobin and form a thicker layer of oxymyoglobin under the surface; this fact translates into a redder meat after oxygenation [26]. However, in extended ageing, the residual oxygen can oxidize oxymyoglobin and deoxymyoglobin, leading to the formation of a brownish layer (metmyoglobin). In essence, as *post mortem* time increases, the reduction ability of muscle decreases and, consequently, the formed metmyoglobin cannot be converted back, giving rise to meat discoloration and brownish color [27,32]. It is known that myoglobin content and reduction activity are muscle and species specific [33].

The implications of ageing process in meat quality have been extensively studied in beef [34], in pork [35] and in sheep meat [36], but only few studies have been performed in horse meat in which, again, differences in animal age and breed are considerable [23,37,38,39,40,41].

Considering the variability of the results obtained and the numerous sources of variation, specific conditions to maximize ageing positive impacts in horse meat are far from being understood and, therefore, further studies are needed. In this regard, the aim of the present work is to study the effect of ageing time (0, 7, 14 and 21 days) in meat quality parameters. This knowledge is necessary to contribute to the recommendation of a certain ageing period, based on quality parameters, for the horse meat industry. Moreover, a thorough characterization of fatty acid (FA) composition of two horse fat tissues, muscle and subcutaneous (SC), has been reported.

## 2. Materials and Methods

### 2.1. Animal Handling

Ten Hispano-Bretón horses (five females and five males) were reared in a commercial farm under grazing conditions while suckling their mothers from birth (May–June 2017) until weaning (6–8 months of age). Then, they continued grazing until 11–13 months of age, when they were moved to a commercial feedlot and finished for 100–120 days on a high-grain diet and straw ad libitum. Concentrate was composed by barley, soybean hulls, molasses, palm oil and salts (13.3% protein, 2.70% fat and 7.60% fiber).

Horses were slaughtered in a commercial abattoir, following the specifications in the European legislation [42], at 15–17 months of age. More details about the experimental design have been previously described [43].

The average carcass weight was of 246 ± 14.0 kg (250 ± 15.0 kg for females and 242 ± 12.5 kg for males). All carcasses were classified as U (conformation) and 2 (fat cover) according to the community scale for the classification of carcasses of adult bovine animals [44] as there is no specific classification system for horses at the EU level.

### 2.2. Experimental Design and Sampling

A full-randomized block design was used considering slaughter day as a blocking factor. Fat tissue was the main factor in the study of the variability of the FA composition of non-aged horse meat samples, and ageing time when assessing the variability of meat quality parameters. In both studies, the loin was the experimental unit. Animal sex, carcass side and carcass weight were distorting variation sources controlled by the experimental design in order to minimize the residual variation. For assessing the effect of ageing time on meat quality parameters a split plot design was used where ageing time levels were randomly allocated to different individual steaks obtained from the loin of each carcass. In this case, the experimental unit (loin) was considered as a plot and the steaks were the subplots (sampling units) in which ageing time effect was assessed.

Two horses (female and male) were slaughtered per week during five consecutive weeks. After 48 h *post mortem* at 4 °C (day 0), both right and left rib sections were removed from carcasses and transported to the laboratory under refrigerated conditions (total number of loins = 20). The loin, longissimus thoracis and lumborum (LTL) muscle, was excised, trimmed of visible adipose and connective tissues and cut into 10 consecutive steaks (1.5 cm thick) from the thoracic side. The first two steaks were trimmed, divided in three similar portions, vacuum packed and frozen (−80 °C) for chemical and FA composition and myoglobin content determinations, respectively. A portion of SC fat (20–30 mg) taken from the thoracic region of the loin was also vacuum packed and frozen at −80 °C for FA profile determination. The following four steaks were vacuum packed and randomly assigned to an ageing time: 0, 7, 14 or 21 d for instrumental color, cooked loss and instrumental texture determinations. The same procedure was used for the last four steaks used for pH measurements. Ageing was performed in a walk-in cooler (4 ± 1 °C) and without illumination. After instrumental color was determined (unpacked and bloomed samples), the steaks were vacuum packed again and frozen at −80 °C for further cook loss and instrumental texture determinations.

### 2.3. Chemical and Fatty Acid Composition

Chemical and FA compositions were determined only in non-aged steaks as not relevant compositional changes were expected within 21 d of ageing. Standard procedures were used for dry matter [45], crude protein [46], ether extract [47] and ash [48] determinations.

For SC fat analysis, 50 ± 1 mg of middle layer adipose tissue were freeze-dried and directly methylated with sodium methoxide (Methanolic-Base, 0.5 N; Supelco Inc., Bellefonte, PA, USA) using 1 mL of internal standard (4 mg/mL of methyl ester (ME) 23:0; Nu-Check Prep. Inc., Elysian, MN, USA). For muscle tissue, total lipids were extracted from 1 g of freeze-dried horse meat with chloroform-methanol (2:1, *v*/*v*) [49]. Lipid aliquots of 10 mg were methylated using anhydrous methanol containing 2% H_2_SO_4_ [16]. Prior to methylation, 1 mL of internal standard (1 mg/mL of 23:0 ME) was added. For both tissues, muscle and SC fat tissues, FAMEs were analyzed using a 7890A gas chromatograph (GC) with flame ionization detector (Agilent Technologies, Madrid, Spain) coupled to a 7693 automatic injector (Agilent Technologies). Separation was carried out using a SP-2560 column (100 m, 0.25 mm i.d. and 0.20 µm film thickness; Supelco. Bellefonte, PA, USA) and following the conditions described by Kramer et al. [50]. Peak identification was performed using commercial reference standards (#463 and #603 mixtures, individual 21:0, 23:0 FAMEs and a CLA mixture #UC-59 M composed by 9*c*,11*t*-/8*t*,10*c*-/11*c*,13*t*-/10*t*,12*c*-/8*c*,10*c*-/9*c*,11*c*-/10*c*,12*c*-/11*c*,13*c*-/11*t*,13*t*-/10*t*,12*t*-/9*t*,11*t*-/8*t*,10*t*-18:2 obtained from Nu-Chek Prep Inc., Elysian, MN, USA; BCFA containing bacterial mixture purchased from Matreya, Pleasant Gap, PA, USA), confirmed using FAME fractions obtained from silver-ion solid phase extraction glass cartridges [51,52], and following retention times and elution orders reported in the literature [5,50,53]. Chromatographic areas were corrected according to theoretical response factors [54] and internal standard was used to calculate quantitative data (mg per g of SC fat tissue and mg per 100 g fresh meat). Then, FAME contents were expressed as percentages (%). In general, FAMEs representing below 0.05% were excluded to reduce the size of tables, except for minor FAMEs of particular nutritional interest.

### 2.4. pH Measurements

Triplicate pH measurements were taken in each steak at each ageing time (0, 7, 14 and 21 d) using a portable pH meter (HI99163, Hanna Instruments, Smithfield, RI, USA) equipped with a penetrating glass electrode (FC232D, Hanna Instruments, USA).

### 2.5. Myoglobin Content Determinations

Total myoglobin content was measured in non-aged steaks as not relevant changes were expected over ageing time. The method by Faustman and Philips [55], with minor modifications was followed. Previously thawed (4 °C, overnight) and minced (domestic grinder) 5 g of meat were homogenized in iced cold sodium phosphate buffer and set aside for 1 h (4 °C, darkness). Samples were then centrifuged (32,000× *g*) for 45 min and filtered through a Whatman filter paper (n °1, Whatman PLC, UK). Absorbance was read at 525 nm in a UV 1280 spectrophotometer (Shimadzu Corporation, Japan) and the total myoglobin concentration was estimated applying the extinction coefficient of 7.6 mM^−1^ cm^−1^ as proposed by Bowen [56].

### 2.6. Instrumental Color Measurements

For instrumental color measurements, color in non-aged steaks was measured after they were cut, covered with an oxygen-permeable polyvinylchloride film (oxygen permeability of 580 mL/m^2^/h) and exposed to air for 1 h (4 °C). The rest of the color measurements were taken when the corresponding ageing period was reached. As for 0 d, steaks were unpacked, covered with film and exposed to air for 1 h (4 °C). A Minolta CR-200 colorimeter (Konica Minolta, Japan) with a D65 illuminant and a 10 ° visual angle was used to measure L* (lightness), a* (redness) and b* (yellowness) values according to CIELAB color space [57]. Five spectral readings per sample were taken in different parts of the steaks. From these measurements, two additional color parameters were calculated: hue angle (h*), which defines color = arctangent (b*/a*) and chroma or saturation index (C*) = (a*^2^ + b*^2^)^1/2^ (AMSA, 2012).

### 2.7. Cook Loss Determinations

Steaks were thawed overnight (4 °C) and after 1 h at room temperature, they were weighted, individually introduced in open plastic bags and cooked in a water bath at 80 °C (SV Thermo, Orved, Italy) until they reached an internal temperature of 71 ± 0.1 °C, monitored by temperature probes (TFA-301040, TFA, Germany). Cooking was performed in batches of 8 steaks, which were randomly chosen (2 sets of 4 ageing times). Then, steaks were removed from the water bath and cooled at room temperature for 1 h. Excess moisture was eliminated and samples were reweighted. Cook loss was determined calculating the weight difference between raw and cooked steaks [58].

### 2.8. Instrumental Texture Measurements

For instrumental texture measurements, 8 cuboids of approximately 1 × 1.5 × 1 cm^3^ were cut from each cooked steak and parallel to the muscle fibers. Maximum Warner-Bratzler shear force (WBSF) of each cuboid was measured using a TA-XT2i texture analyzer (Stable Micro Systems, UK) connected to an IBM-compatible Foxen computer, with an AutenticAMD-K6 ™ 3D microprocessor. The ‘Texture Expert’ software, version 1.22 for Windows (Stable Micro Systems, Surrey, UK) was used. The texture analyzer was equipped with a WBSF device (cutting blade at a constant speed of 1.70 mm/s and a load cell of 300 N).

### 2.9. Statistical Analysis

Analyses were conducted using IBM-SPSS Statistics Software (Version 25.0, IBM Corporation, Chicago, IL, USA). Normality and homoscedasticity of the variables were checked and pH values were log transformed.

The general linear model (GLM) of ANOVA was used to determine, separately, the significant differences in the FA composition between fat tissues (muscle and subcutaneous fat) of non-aged horse meat samples, and in the instrumental color (L*, a*, b*, C*, h*), pH, cook loss and WBSF measurements of horse meat samples among ageing times (0, 7, 14 and 21 d). The GLMs included the corresponding main factor and the controlled distorting variation factors (animal sex and carcass side) as fixed effects, and carcass weight as a covariate. Slaughter day was also included as a random effect because this blocking factor was a simultaneous distorting factor of uncontrolled variation coming from at least individual animal, feeding, transport or slaughter conditions. Moreover, GLMs included binary interactions between all factors.

Eta square (*ɳ*^2^) was used for effect size estimation and Fisher’s least significance difference test of estimated marginal means was used for pairwise comparisons among ageing time levels. Three significant figures were used to express data and significance was declared at *p* ≤ 0.05.

## 3. Results and Discussion

### 3.1. Chemical and Fatty Acid Composition

Chemical composition (%) and muscle FA composition in absolute basis (mg/100 g of raw meat) are reported in Table 1. Mean moisture (75.3%) was comparable to values described in the literature for 15–16 month old horse loins [7,59,60]. As expected, mean protein content (20.4%) was also in the range of values reported in the literature for different horse breeds, ages and production systems (19–22%; [7,19,21,23,59,60,61,62,63]). The muscle fat content obtained in the present study (3.31%) was comparable to other studies where horses were extensively reared, finished on concentrates and slaughtered at similar ages [7,19,60]. Ash content in horse meat has been reported to vary due to the age of animals [64] while the mineral content of feedstuffs may also have an influence [64]. The results obtained in the present study (1.03%) were in line with meat from horses slaughtered at similar age [7,19,59].

In terms of FA composition, in absolute basis (Table 1), wide ranges were observed while these differences were substantially reduced in percentage basis (Table 2, Table 3 and Table 4). Variable lipid content in horse meat has been previously reported, depending primarily on animal-related (i.e., breed, age, feeding) but also methodology-related (i.e., fat extraction procedure) factors [3].

Considering the saturated fraction of muscle lipids (Table 1 and Table 2), the obtained results (333–1743 mg/100 g of meat; 38.9%) were in accordance with those reported in horse meat [3]. Palmitic acid (16:0) was the major FA (29.3%) followed far behind by stearic (18:0) and myristic (14:0) acids (4.79% and 3.65%, respectively). The rest of minor SFA altogether constituted approximately only 1% of the total SFA.

Traditionally, BCFAs have not been discussed in horse meat studies since they are primarily associated to ruminant derived products due to their microbial origin [14]. However, they were described in tissues of other herbivores, such as rabbits, with microbial fermentation in the hindgut [65], which is also the case of horses. In this sense, recent studies have reported low contents of BCFAs in horse meat (6.25 mg/100 g of meat) [5,61], in line with the present study (4.98 mg/100 g of meat). The major BCFA in these studies was *iso*-16:0 and this is also supported by the present results (42% of total BCFA) (Table 1 and Table 2).

Regarding monounsaturated FAs (MUFA), the absolute content ranged between 380 and 1877 mg/100 g meat, comprising 42.9% of total FAMEs (Table 1 and Table 3). This value locates among those reported in the literature for extensively reared (14.6%) and concentrate fed (50.2%) horses slaughtered at different ages [3]. *Cis(c)*-MUFAs represented 99.7% of MUFAs, with oleic (9*c*-18:1), palmitoleic (9*c*-16:1) and ascleptic (11*c*-18:1) acids being the most abundant. *Trans (t)*-MUFAs have not been generally reported in horse meat studies [6,8,62,66,67] as it is known that their content is low compared to ruminant products. The low accumulation of these FAs in equids is related to their digestive physiology [12,13]. In the present study, *t*-MUFA accounted only for 0.32% of total MUFA, with elaidic acid (9*t*-18:1) being the major *t*-MUFA. CLAs are also minor in horse meat compared to ruminants, as they are primarily produced from PUFA by the action of rumen microbiota. However, as previously discussed, a low accumulation of these compounds is possible. In the present study, the total CLA represented 0.12% and similar percentages have been reported by He et al. [6] in 3 year old Breton horses fattened for 12 months (0.15%) and by Belaunzaran et al. [61] in fattened and suckling crossbred horses (0.11%). In contrast, higher proportions have reported Juarez et al. [62] in 24 month old Hispano-Bretón and Burguete horses (0.42%) and by Lanza et al. [7] in 18 month old Sanfratello and Haflinger horses (0.46%), which could well be related to the higher muscle fat content but also to the overestimation of CLA, taking into account that 21:0 elutes in the CLA region (GC technique). Rumenic acid (9*c*,11*t*-18:2) was the major CLA isomer and in relation to non-conjugated (NC) dienes and trienes, their contribution was minor (<0.1%) as reported by Belaunzaran et al. [61].

In general, horse meat has been described as rich in PUFAs (specially in essential linoleic (LA, 18:2n-6) and linolenic (LNA, 18:3n-3) acids) because, as discussed earlier, the FA absorption in the equine digestive system happens before the fermentative chambers, allowing an efficient absorption and deposition of dietary PUFAs in horse products with little transformation [3]. An average PUFA content of 345 mg/100 g of meat was found, accounting for 15.0% (Table 1 and Table 4). This percentage was low compared to other studies reporting values from 15.6% in 3 year old concentrate-fed Breton horses [6] to 46% in 2 year old grass fed Galician Mountain horses [66]. The low PUFA content related to the low n-6 content, especially LA that is the major n-6 PUFA (86% of total n-6 PUFA). On average, LA accounted for 10.1% of total FAMEs, a percentage that is below those usually reported in horses slaughtered at similar ages and managed under semiextensive (20.9–21%, [7]; 16.6–18.9%, [63]) systems. However, similar values to the present study were obtained by Lorenzo et al. [68], in a study performed with semi-extensively reared 18 month old Galician Mountain × Hispano-Bretón horses (10.1%). Thus, the high-grain finishing period of the animals probably caused a remarkable decrease in LA percentage in favor of MUFAs. Regarding the rest of n-6 PUFAs, arachidonic acid (20:4n-6) was the second most abundant, in good agreement with previous works [5,7,18,66,67,68].

The capacity of horses to absorb dietary PUFAs before microbial hydrogenation has been related to the high accumulation of n-3 PUFAs from pastures (rich in LNA, 18:3n-3) [3]. More recently, as reviewed by Sahaka et al. [9], a specific pancreatic enzyme (pancreatic lipase related protein 2) hydrolyzing the LNA esterified in plant galactolipids has been described to be uniquely present in horse, contributing to LNA deposition in horse tissues. In the scientific literature, n-3 values from 1.53% in 36 month old Hispano-Bretón horses finished on concentrate for 12 months [18] up to 24.4% in 9 month old extensively reared Galician Mountain horses [69] have been reported, being diet the main source of variation and LNA the main responsible for those elevated n-3 contents. In the present study, an average value of 3.19% of n-3 PUFA (78 mg/100 g of meat) was observed, evidencing that the high-grain finishing limited the n-3 PUFA deposition. On average, LNA accounted for 2.26% and the next major n-3 PUFAs were 22:5n-3 (docosapentaenoic acid, DPA), 20:5n-3 (eicosapentaenoic acid, EPA), 22:6n-3 (docosahexaenoic acid, DHA) and 20:3n-3. These four FAs, although being cell membrane components, are seldom reported in horse meat studies.

Overall, obtained n-3 percentage was similar to the ones typically described in non n-3 enriched chicken (3.03%, [70]) or rabbit (3.40%, [71]) and higher than non n-3 enriched pork (1.22% vs. 8.94% in 10% flaxseed enriched pork [72]).

Alkenyl moieties from plasmalogenic lipids in horse meat have not received much attention, although they have been studied in other meats [73,74]. The explanation for this may lie in the technical difficulties to resolve these compounds [15]. Under the conditions of the present study (acid-catalyzed methanolysis), these were detected as the sum of dimethylacetals, alk-1-enyl methyl ethers and fatty aldehydes. An average content of 60.3 mg/100 g of meat was found, in line with other horse meat studies [15,61]. These findings should not be diminished and deserve further research due to the increasing reports concerning the biological activities of plasmalogenic lipids and their relationship with human health [16,17].

The results of the statistical analysis showed several significant differences in the FA content between muscle and SC fat (Table 2, Table 3 and Table 4). SC tissue showed significantly higher SFA content than muscle fat as the former is mainly composed of triacylglycerols, major components of neutral lipids that, in general, exhibit low PUFA and high SFA and MUFA depositions [61]. Conversely, 18:0 showed significantly higher content in muscle than in SC fat as observed by Belaunzaran et al. [5], which justified this phenomenon proving that this FA is mainly esterified in polar lipids [61]. Regarding MUFAs, significant differences were observed in several individual FAs and oleic acid (9*c*-18:1) was found in higher percentages in SC fat. In the literature, higher MUFA contents have been reported in SC compared to muscle fat [5,62,63]. Finally, no difference was observed in total PUFA content between the tissues although most long-chain n-6 and n-3 PUFAs were significantly higher in muscle compared to SC fat. In line with this, Belaunzaran et al. [61] described FA deposition preferences in horse muscle, indicating that all PUFAs were more abundant in polar lipids (phospholipids) except LNA that was preferentially deposited in neutral lipids (triacylglycerols).

### 3.2. Effect of Ageing on Horse Meat Quality Parameters

**Figure 1:**
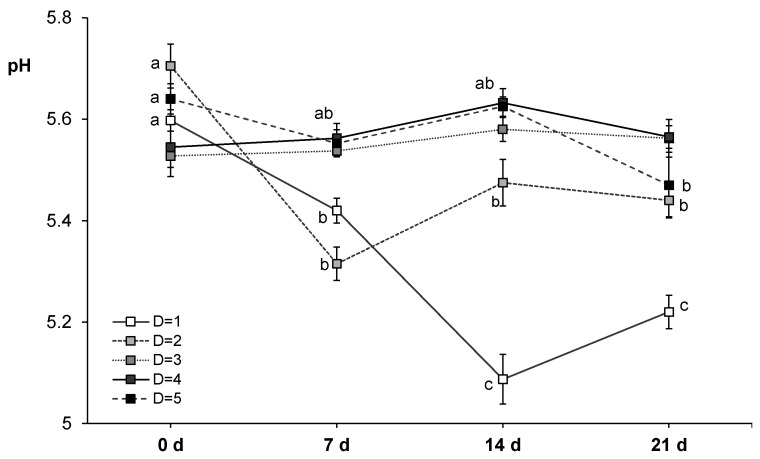
Interaction between ageing time (0, 7, 14 and 21 days) and slaughter day (D; 5 consecutive weeks) for pH measurements in horse longissimus thoracis and lumborum muscle (*n* = 20). Mean values and standard error of the means have been represented. Different letters indicate significant differences (*p* ≤ 0.05) among ageing times.

Ageing time significantly affected all the quality parameters (pH, instrumental color, cook loss, WBSF; Table 5). Additionally, the statistical analysis revealed that slaughter day (five consecutive weeks) and its interaction with ageing time were also significant for some of the quality parameters studied, which could be attributed to other preslaughter factors affecting the oxidation state and color [75,76,77], texture and other quality attributes of meat [78]. However, when the interaction term was statistically significant, it was ordinal for all parameters and its effect size (measured by *ɳ*^2^) was smaller than the main effect (ageing time), except for pH measurements. In this case, the interaction between ageing time and slaughter day evidenced the effect of other uncontrolled preslaughter factors (Figure 1).

#### 3.2.1. Measurement of pH

Initial pH value measured at 48 h *post mortem* (5.60 ± 0.09) was similar to that reported by others [23,59,60,62] in horse loins of Hispano-Bretón, Galician Mountain, Burguete and Jeju breeds, respectively. This would mean that a normal acidification was achieved during *post mortem* metabolism. In the literature, defects coming from abnormal *post mortem* metabolism have not been reported in horse meat [22,79]. The reason of pH value being slightly lower than the one typically registered in beef [80,81] could be related to the higher glycogen content reported in horse meat compared to beef [79], after rigor completion it would store more residual glycogen and give lower pH values. During the ageing period, in general, pH values decreased from 0 to 7 d (mean value of 5.48) and, then, stayed stable until the end of the experiment (21 d), except for the first slaughter day that values continued decreasing until 14 d (Figure 1). The reason for this behavior is not immediately apparent but it is likely attributed to other non-controlled factors. Related to pH decrease under vacuum, growth of lactic acid bacteria (LAB) has been reported to be the main responsible for pH decrease in refrigerated meats (pH < 5.8; [32]). Gomez and Lorenzo [38] reported a slight increase of LAB in vacuum packed horse meat during the first 7 d of ageing, without developing spoilage odors that could be the reason for the pH decrease during vacuum ageing. In contrast, Seong et al. [23] observed an increase of pH until 20 d of ageing, whereas others reported pH increases during the first 2 weeks of ageing [38,40].

#### 3.2.2. Myoglobin Content and Instrumental Color

Myoglobin content in meat is highly relevant as it determines meat color via its four chemical forms [82]. In particular, horse meat has been described as rich in myoglobin compared with other meats [83]. The chemistry of myoglobin is species specific [33]. In this sense, a high ability of oxygen to combine with the red oxymyoglobin and convert to brown metmyoglobin has been described in horse meat, affecting to the shelf life of meat [84]. The results obtained in the present study (3.47 mg/100 g of meat) were comparable with values reported in extensively reared horses slaughtered at similar age [63,84], but higher than those found in animals slaughtered at earlier ages [21], which is in accordance with what Badiani and Manfredini [83] indicated, that myoglobin content in horses increases during the first two years of life.

**Figure 2:**
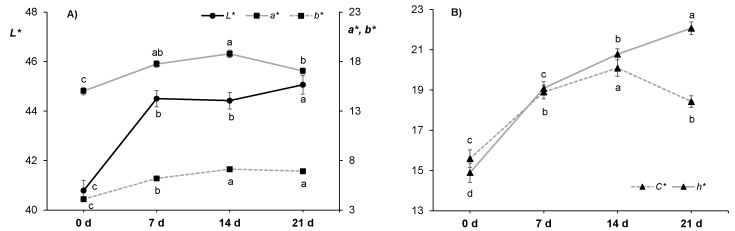
Effect of ageing time (0, 7, 14 and 21 days) on instrumental color measurements in horse longissimus thoracis and lumborum muscle (*n* = 20): (**A**) L* (left axis), a* and b* (right axis), and (**B**) C* and h*. Different letters indicate statistically significant differences (*p* ≤ 0.05).

Regarding instrumental color parameters (Figure 2), lightness (L*) values of the loin (40.8–45.1) were consistent with the literature [21,40]. However, others have reported lower (28–35 [62]; 35–37 [23]; 32–37 [85]) and higher (49–52 [84]) L* values in horses slaughtered at a similar age. In the present study, lightness significantly increased during the ageing period (*p* ≤ 0.001) with no changes among 7 and 14 d (Figure 2A). Lightness values reported during vacuum ageing in horse meat studies showed diverse patterns. For instance, Lorenzo and Gomez [40] and Seong et al., [23] did not observe any difference during the first 14 d and 30 d of ageing, respectively, while Ruiz [41] reported increased loin L* values when 13 month old horses were fed linseed enriched concentrate compared to regular concentrate. Overall, according to literature, L* values are not supposed to vary much during *post mortem* storage, showing only slight increases [86], which is due to changes in light scattering associated to muscle fibers shrinkage and fluids expulsion to the extracellular space [87].

Redness (a*) values (15.0–18.8; Figure 2A) were in good agreement with those reported by others in horse loins [23,38,40,62], although others reported lower values [21,84]. These variations in redness could be attributed to the total myoglobin content [63] that depends primarily on animal age [83]. Regarding the ageing time, redness was the highest at 14 d (18.8) and the lowest (15.0) in non-aged loins, whereas at 7 d and 21 d intermediate values were observed. A significant increase in redness from 0 to 14 d in vacuum aged meat was also observed by Lorenzo and Gomez [40] while Seong et al. [23] reported a continuous increasing trend up to 30 d.

Yellowness (b*) values (4.09–7.12; Figure 2A) were similar to some [20,21,23] but lower compared to other horse meat studies (12–14 [38]; 10–14 [40]; 8–15 [84]) and these variations could be attributed to differences in muscle fat content and slaughter age of horses [63]. In the present study, during ageing time, b* values increased from 4.09 (0 d) to 7.12 (14 d) while no difference was found between 14 and 21 d. Gomez and Lorenzo [38] and Lorenzo and Gomez [40] also observed an increase in yellowness during vacuum storage that plateaued at 7 and 10 d, respectively, while Seong et al. [23] reported an increase in b* values over during 30 d of ageing.

**Figure 3:**
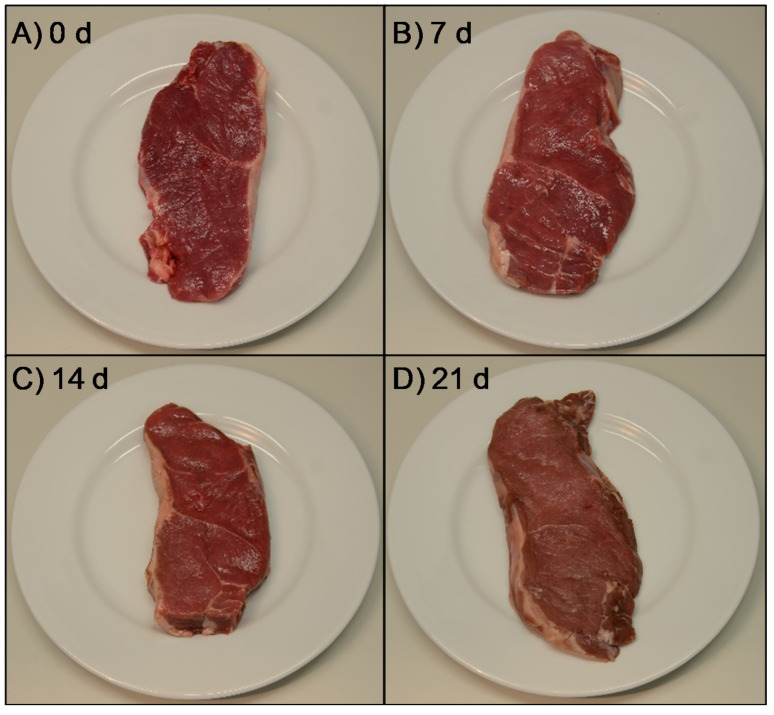
Representative photographs of non-aged (**A**) and aged horse longissimus thoracis and lumborum steaks ((**B**) 7 days, (**C**) 14 days and (**D**) 21 days).

In terms of calculated chroma (C*) and hue (h*) values, the decrease observed in redness but not in yellowness between 14 and 21 d resulted in a significant increase of hue value from 20.8 to 22.1 (Figure 2B) in the yellow (+b*) direction of the CIELAB color space. This happened together with a decrease in chroma (C*) from 20.1 to 18.4, being recognized as a more grey or dull color (dull yellow) perceived as brown. This would indicate that horse meat started to turn brownish between 14 and 21 d of ageing due to metmyoglobin formation [28,86]. The color changes during ageing period are well illustrated in Figure 3, where a representative photograph of each ageing time is shown. It is worth commenting that samples aged for 21 d were visually evaluated by 120 consumers and these samples obtained the worst acceptability scores [43]. After 14 d of ageing, the visually apparent color shift to brownish could be the reason why consumers rejected the meat aged for 21 d, as the cherry-red color that they associate with freshness was not present [88].

#### 3.2.3. Cook Loss and Instrumental Texture

**Figure 4:**
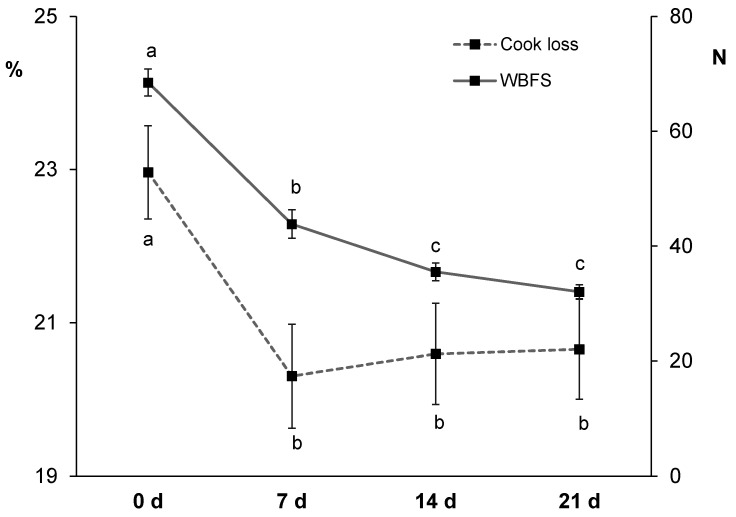
Effect of ageing time (0, 7, 14 and 21 days) on cook loss (left axis) and Warner-Bratzler shear force (WBSF; right axis) in horse longissimus thoracis and lumborum muscle (*n* = 20). Different letters indicate statistically significant differences (*p* ≤ 0.05).

It is known that cooking contributes to meat texture changes, while moisture and cook losses happen in a time and temperature dependent manner [89]. Cook loss values ranged between 20.3 and 23.0% (Figure 4). The value obtained in non-aged horse meat (23%) was similar to those values reported by others in cooked horse loins [23,39]. Values decreased from non-aged to 7 d aged meat (20.3%) and then stayed constant. It is known that ageing affects water distribution and mobility in meat [90] but contradictory results have been reported in the literature regarding cook loss data. In some studies, an increase in cook loss with ageing has been observed as a result of a weakened muscle structure unable to trap or retain water [87] while in others a decrease has been reported as a result of a lower initial water content related to a pH drop [91,92]. The second statement seem to better explain the results of the present study as a decrease in both pH and cook loss happened between 0 and 7 d of ageing (Figure 1 and Figure 4). In the scientific literature, Seong et al. [23] did not observe any differences in cook loss during 30 d ageing whereas Kaic et al. [39] reported an increase in cook loss from 14 to 28 d of horse meat ageing. Overall, only few studies have reported cook loss data in horse meat and the variability of results is considerable.

Regarding instrumental texture measured in cooked horse meat, values ranged between 32.1 and 68.5 N (Figure 4). The WBSF value in non-aged horse loin (68.5 N) was higher than values reported in the literature (40 N [23]; 31 N [37]) but close to values reported in 4–6 d aged meat from 18 month old Sanfratellano and Haflinger horses (56.5 N [7]), in 2 d aged meat from 10 year old horses (63 N [22]), and in non-aged meat from 13 and 26 month old Galician Mountain × Burguete horses (52.5 N [41]). In the present study, as expected, WBSF values decreased significantly from 0 (68.5 N) to 14 d (35.5 N), with no additional significant changes (32.1 N at 21 d). Considering the WBSF-dependent classification proposed by Shackelford, Morgan, Cross and Savell [93] for beef loin as there is no homologous classification for horse meat, non-aged meat would be classified as ‘tough’, 7 d aged meat as ‘intermediate’, and 14 d and 21 d aged meat as ‘tender’. In essence, due to expected *post mortem* proteolysis [30], ageing period improved meat tenderness, especially during the first two weeks. Similarly, Seong et al. [23] observed a decrease in WBSF values during the first 10 d of ageing (getting classified as ‘very tender’) in meat from 28 month old Jeju horses. Some other horse meat studies have reported earlier tenderization; for instance, Della Malva et al. [37] reported the most tender horse meat after only 3 d of ageing and they did not perceive any additional changes thereafter. Others have reported significant improvement of WBSF values after 4 and 8 d of ageing depending on animal feeding and age [41].

## 4. Conclusions

The fatty acid composition of Hispano-Bretón horse meat characterized in the present study indicated that the high-grain finishing period may have limited the deposition of polyunsaturated fatty acids in tissues, especially of n-3, compared to values typically described in grazing horses. However, obtained n-3 polyunsaturated fatty acid contents were still higher than those generally reported in ruminant derived products although LNA was preferentially deposited in backfat compared to muscle fat, confirming the potential of horse as an interesting red meat alternative in terms of its nutritional quality. The present study highlights that the ageing process affects horse meat quality parameters that are very relevant for the consumer. Visual properties started to deteriorate after 14 days of ageing and the improvement in tenderness also happened during the first 14 days, although meat could be considered as ‘intermediate tender’ after the first week of ageing. From the results obtained, the optimum ageing time can be established between 7 and 14 days for Hispano-Bretón horse meat; longer periods would not be justified from the quality point of view nor from the economic point of view. All in all, further research is required in order to standardize *post mortem* practices so as to obtain homogeneous final horse meat quality.

## Figures and Tables

**Table 1:** Chemical (%) and fatty acid (absolute basis: mg/100 g of meat) composition of horse longissimus thoracis and lumborum muscle (*n* = 20).

	Mean	Min	Max	SEM
**Chemical composition**				
Moisture	75.3	72.9	78.1	0.3
Crude protein	20.4	19.1	22.3	0.2
Fat (ether extract)	3.31	1.87	5.13	0.19
Ash	1.03	0.750	1.17	0.02
**Fatty acid composition**				
Total FAME	2427	893	4238	199
SFA	956	333	1743	85
BCFA	4.98	2.32	7.97	0.35
MUFA	1054	380	1877	93
*cis*-MUFA	1051	378	1872	92
*trans*-MUFA	3.32	1.07	5.42	0.29
CLA (18:2)	2.75	1.42	4.36	0.21
NC-dienes (18:2)	1.40	0.814	2.91	0.11
Trienes (18:3)	0.522	0.105	1.14	0.05
PUFA	345	147	546	22
n-6	266	113	390	15
18:2n-6	231	96	350	14
n-3	78	25	177	8
18:3n-3	56.1	15.6	154	8
DMA	60.3	26.6	84.7	3.3

Min, minimum value; Max, maximum value; SEM, standard error of the mean. FAME, fatty acid (FA) methyl esters; SFA, saturated FA; BCFA, branched-chain FA; MUFA, monounsaturated FA; CLA, conjugated linoleic acids; NC, non-conjugated; PUFA, polyunsaturated FA; DMA, dimethylacetals (sum of DMA, AME (alk-1-enyl methyl ethers) and ALD (fatty aldehydes).

**Table 2:** Effect of fat tissue (muscle and subcutaneous fat) on the saturated and branched-chain fatty acid composition of horse meat in percentage basis (%) (*n* = 20).

	Muscle				Subcutaneous		*p*-Value
	Mean	Min	Max	SEM	Mean	SEM	
SFA	38.9	36.8	43.8	0.3	39.5	0.5	0.383
12:0	0.206	0.151	0.256	0.006	0.224	0.008	0.079
14:0	3.65	3.00	4.58	0.09	4.33	0.09	<0.001
15:0	0.274	0.186	0.429	0.015	0.410	0.017	<0.001
16:0	29.3	26.8	31.3	0.3	29.9	0.4	0.256
17:0	0.357	0.238	0.489	0.015	0.505	0.020	<0.001
18:0	4.79	4.38	5.50	0.09	3.85	0.14	<0.001
20:0	0.0590	0.0450	0.0700	0.0020	0.0496	0.0017	<0.001
22:0	0.0665	0.0370	0.106	0.0039	0.0138	0.0018	<0.001
BCFA	0.211	0.176	0.260	0.006	0.285	0.012	<0.001
*i*-16:0	0.0890	0.068	0.107	0.0023	0.0844	0.0037	0.297

Min, minimum value; Max, maximum value; SEM, standard error of the mean. SFA, saturated fatty acids (FA); BCFA, branched-chain FA.

**Table 3:** Effect of fat tissue (muscle and subcutaneous fat) on the monounsaturated fatty acid composition of horse meat in percentage basis (%) (*n* = 20).

	Muscle					Subcutaneous		*p*-Value
	Mean	Min	Max	SEM		Mean	SEM	
MUFA	42.9	37.6	47.7	0.6		44.3	0.4	0.074
*cis*-MUFA	42.8	37.4	47.5	0.6		44.2	0.4	0.076
9*c*-14:1	0.412	0.297	0.511	0.013		0.389	0.016	0.295
7*c*-16:1	0.182	0.138	0.272	0.008		0.328	0.014	<0.001
9*c*-16:1	8.09	5.602	9.693	0.27		7.58	0.23	0.174
9*c*-18:1	31.4	27.4	34.1	0.4		32.7	0.4	0.025
11*c*-18:1	1.93	1.57	2.22	0.04		1.51	0.03	<0.001
13*c*-18:1	0.0965	0.0740	0.119	0.0027		0.0531	0.0017	<0.001
11*c*-19:1	0.0675	0.0491	0.0880	0.0027		0.0738	0.0042	0.201
11*c*-20:1	0.335	0.283	0.416	0.008		0.470	0.016	<0.001
*trans*-MUFA	0.136	0.101	0.237	0.006		0.149	0.005	0.143
9*t*-18:1	0.0970	0.0680	0.117	0.0026		0.0625	0.0026	<0.001

Min, minimum value; Max, maximum value; SEM, standard error of the mean. MUFA, monounsaturated fatty acids. (FA).

**Table 4:** Effect of fat tissue (muscle and subcutaneous fat) on the conjugated and non-conjugated diene, triene, polyunsaturated fatty acid and dimethylacetal composition of horse meat in percentage basis (%) (*n* = 20).

	Muscle				Subcutaneous		*p*-Value
	Mean	Min	Max	SEM	Mean	SEM	
CLA (18:2)	0.118	0.0884	0.198	0.007	0.0931	0.0050	0.008
9*c*,11*t*-	0.0535	0.0394	0.0712	0.0022	0.0581	0.0043	0.318
NC-dienes (18:2)	0.0605	0.0372	0.1002	0.0033	0.0475	0.0030	0.008
Trienes (18:3)	0.0220	0.0073	0.0371	0.0016	0.0263	0.0020	0.099
PUFA	15.0	11.1	21.6	0.7	15.6	0.5	0.455
20:3n-9	0.0263	0.0174	0.0441	0.0019	0.0125	0.0014	<0.001
n-6	11.7	8.76	19.38	0.7	12.5	0.6	0.424
18:2n-6	10.1	7.55	16.6	0.6	12.0	0.5	0.026
20:2n-6	0.206	0.141	0.335	0.012	0.281	0.012	<0.001
20:3n-6	0.240	0.124	0.387	0.015	0.0338	0.0022	<0.001
20:4n-6	0.946	0.491	1.74	0.071	0.0631	0.0053	<0.001
22:4n-6	0.0705	0.0381	0.160	0.0067	0.0194	0.0021	<0.001
22:5n-6	0.0470	0.266	0.793	0.0054	ND	-	-
n-3	3.19	2.05	5.31	0.21	3.18	0.23	0.975
18:3n-3	2.26	1.11	4.14	0.19	2.98	0.21	0.018
20:3n-3	0.110	0.0720	0.188	0.007	0.106	0.008	0.714
20:5n-3	0.125	0.0480	0.296	0.0134	0.0119	0.0010	<0.001
22:5n-3	0.503	0.266	0.793	0.027	0.0561	0.0034	<0.001
22:6n-3	0.115	0.0460	0.187	0.0084	0.0120	0.0010	<0.001
n-6/n-3	3.97	2.06	8.77	0.38	4.30	0.45	0.565
P/S	0.386	0.284	0.587	0.020	0.399	0.016	0.609
DMA	2.63	1.531	3.472	0.11	ND	-	-

Min, minimum value; Max, maximum value; SEM, standard error of the mean. CLA, conjugated linoleic acids; NC, non-conjugated; PUFA, polyunsaturated fatty acids; DMA, dimethylacetals (sum of DMA, AME (alk-1-enyl methyl ethers) and ALD (fatty aldehydes)); ND, not detected.

**Table 5:** Statistical parameters on the effect of ageing time (0, 7, 14 and 21 days), slaughter day (5 consecutive weeks) and ageing time*slaughter day on pH, instrumental color (L*, a*, b*, C*, h*), cook loss (%) and Warner-Bratzler shear force (N) measurements in horse longissimus thoracis and lumborum muscle (*n* = 20).

	AT		D		AT*D	
	*p*-Value	*ɳ* ^2^	*p*-Value	*ɳ* ^2^	*p*-Value	*ɳ* ^2^
pH	< 0.001	0.583	< 0.001	0.758	< 0.001	0.796
L*	< 0.001	0.884	0.001	0.348	< 0.001	0.586
a*	< 0.001	0.665	0.015	0.251	0.319	0.254
b*	< 0.001	0.858	0.067	0.185	0.025	0.394
C*	< 0.001	0.718	0.024	0.230	0.230	0.278
h*	< 0.001	0.923	0.041	0.207	< 0.001	0.558
Cook loss	0.007	0.246	0.004	0.297	0.577	0.200
WBSF	< 0.001	0.926	< 0.001	0.684	0.114	0.321

AT, ageing time; D, slaughter day; *ɳ*^2^, eta square; WBSF, Warner-Bratzler shear force.

## Data Availability

The data presented in this study are available on request from the corresponding author.
